# Items

**Published:** 1883-07

**Authors:** 


### Items.

Dr. S. V. Clevenger, of Chicago, has been appointed Pathologist to Cook County Hospital for the Insane, near this city. The doctor has been a laborious worker in the neurological and psychiatric field for many years past, and has been well known as a contributor to these pages. He now abandons the practice of medicine, and enjoys the long coveted opportunity of devoting his inquiries exclusively to the study of pathology of the nervous system. We shall often hear from him in his new field of labor. Subjoined is a list of his contributions to scientific literature in the past. We publish it that it may be more generally known with what ripe experience and training this newly appointed pathologist enters upon a career where he will have such large opportunities. We unite with his many friends in wishing that, with these, he may accomplish a still more brilliant future.

1. Engineering Instruments of Aluminum. Published in *Van Nostrand's Engineering Magazine*, February, 1874. The use of this metal was suggested in the manufacture of all instruments where bulkiness and lightness were *desiderata.*

2. Construction of a New Mean Noon Sun Dial. *Engineering Magazine*, July, 1874.

Describing an invention of the doctor's, by means of which clock time could be obtained by simple inspection, the dial being self-equating.

3. American Cartography. *Engineering Magazine*, May, 1875.

A description of the sources of information resorted to by map compilers in America.

4. A Treatise on the Methods of U. S. Land Surveying, pp. 200. D. Van Nostrand, New York, Publisher. 1874. Three Editions.

A practical application of astronomy and mathematics.

5. Therapeutic Action of Mercury. Read before the Chicago Biological Society, February 4, 1880. *Chicago Medical Gazette* (Review) Feb. 20, 1880.

6. Mechanical Therapeutics, Chemistry, and Toxicology of Mercury. CHICAGO MEDICAL JOURNAL AND EXAMINER, April, 1880.

7. Examination of Tissues after the Administration of Mercury. Read before the Illinois State Microscopical Society, February 27, 1880. *American Journal of Microscopy*, June and July, 1880.

The foregoing papers embody experiments to sustain Dr. Clevenger's theory that all mercurials act mechanically in a state of extremely minute division in the histological channels of the body.

8. Cerebral Topography. *Journal of Nervous and Mental Disease*, October, 1879.

The nomenclature of portions of the brain in Greek, Latin, German, English, French and Italian, with a description of researches into cerebral functions.

9. The Sulcus of Rolando, as an Index to the Intelligence of Animals. *Journal of Nervous and Mental Disease*, April, 1880.

Comparisons of the position of this fissure in the brains of many animals, with the suggestion that it forms a good posterior boundary for the frontal lobe, and that in an ascending scale of intelligence the fissure advances backward.

10. Guide to Post-Mortem Examinations of the Brain. *Chicago Medical Gazette* (Review), March, 1880.

11. Laceration of the Cervix Uteri a Probable Cause of Recurring Abortions. *Gazette*, Jan. 20, 1880.

12. Reports of Cases. *Gazette*, June 5, 1880.

13. *Chicago Medical Gazette* Editorials during 1880.

14. *Chicago Druggist* Editorials during 1881.

15. Book Reviews, *Journal of Nervous and Mental Disease:* Benedikt, Brains of Criminals, Jan. 1880; Richet and Pansch on the Brain, Oct. 1879; Bastian, The Brain as an Organ of the Mind, Jan. 1881.

16. Plan of the Cerebro-Spinal Nervous System. Read before the American Association for the Advancement of Science, Boston, Mass., Aug. 28, 1880. *Journal of Nervous and Mental Disease*, October, 1880.

17. Comparative Neurology. *American Naturalist*, January and February, 1881.

18. Origin and Descent of the Human Brain. Read before the Chicago Academy of Sciences, February 8, 1881. *American Naturalist*, July, 1881.

19. Cerebral Anatomy Simplified. CHICAGO MEDICAL JOURNAL AND EXAMINER, November, 1880.

The four last-mentioned papers defend the theory advanced by the doctor, that the cerebellum of all animals is composed of a number of intervertebral ganglia, more or less fused together, and that all the lobes of the brain are similarly originated, just as the Gasserian ganglion is an intervetebral.

20. The Study of Biology, Its Importance and Tendencies. A lecture delivered before the Chicago Electrical Society, November 21, 1881.

21. Drug Adulterations. *Druggist*, Feb. 1881.

22. Oleate of Mercury. *Druggist*, Jan. 1882.

23. Schmidt on Yellow Fever. Read before the Chicago Biological Society, April 6, 1881. CHICAGO MEDICAL JOURNAL AND EXAMINER, May, 1881.

24. Medical Electricity. Vice-Presidential address to the Chicago Electrical Society, October, 1881. CHICAGO MEDICAL JOURNAL AND EXAMINER, Nov. 1881.

25. Nerve-Cells and their Function. Read before the Illinois Microscopical Society, March 11, 1881. Abstracted (badly) in *Chicago Medical Review*,, March 20, 1881.

26. Nerves and Nerve-Cell Function. Inaugural Thesis, American Neurological Society, New York, June, 1881.

Advancing the idea that the main function of the nerve-cell was histogenetic, and not force-producing.

27. Somatic Physics. American Electrical Society Thesis, December 8, 1880.

28. Probable Branchial Origin of the Thyroid and Thymus Glands. "*Science*" (New York), June 25, 1881. Suggestion that these bodies are rudimentary gills.

29. Hunger the Primitive Desire. "*Science*," Jan. 1881. A philosophical fusion of the sexual and ingestive acts.

30. Contributions to Comparative Psychology. No. I, Instinct and Reason, "*Science*," May 28, 1881; No. II, Origin of Language. "*Science*," July 23, 1881.

31. Artistic Anatomy and the Sciences Useful to the Artist. Opening lecture of a course delivered at the Art Institute during the winter term, 1882-3. CHICAGO MEDICAL JOURNAL AND EXAMINER, February, 1883.

32. Disadvantages of the Upright Position. A lecture delivered before the Chicago University Club, April 1882, and Philadelphia Academy of Sciences, May 23, 1883. *American Naturalist*, July, 1883.

---

HER Royal Highness, Princess Christian of Schleswig-Holstein (Princess Helen of England), has taken a course of lectures in the Kensington Centre Institution, and, passing the examination, she received her diploma as a "nurse." She is the same who translated into the English language the work of her brother-in-law, Prof. Romarch, at Kiel, which he wrote for the first instruction in accidents.

---

THE well known "vegetarian" Wagner, at Basel, died from cancer of the stomach. He taught total abstinence from meat and he lived only on vegetables. He called his way of living the "long-life" way, but he was only 43 years of age when he died.

---

THE *Med. Chir. Central-Blatt* at Vienna, has recently been confiscated on account of an article on the titles of surgeons in this happy country.

